# Supplementary material for: Appetite loss at discharge from acute decompensated heart failure: Observation from KCHF registry
Source: PLoS One. 2022 May 5;17(5):e0267327. doi: 10.1371/journal.pone.0267327 (PMC9071124; doi:10.1371/journal.pone.0267327)
Supplement: S1 Fig — Kaplan-Meier curves according to the degree of appetite loss at discharge for (A) the primary outcome measure (all-cause death), (B) CV death, (C) non-CV death, and (D) HF hospitalization. HR = hazard ratio, CI = confidence interval, CV = cardiovascular, HF = heart failure. (PDF) [file pone.0267327.s001.pdf]

**S3 Fig.** Kaplan-Meier curves according to the degree of appetite loss at discharge for (A) the primary outcome measure (all-cause death), (B) CV death, (C) non-CV death, and (D) HF hospitalization. HR=hazard ratio, CI=confidence interval, CV=cardiovascular, HF=heart failure.

## *(A) Primary outcome measure (all-cause death)*

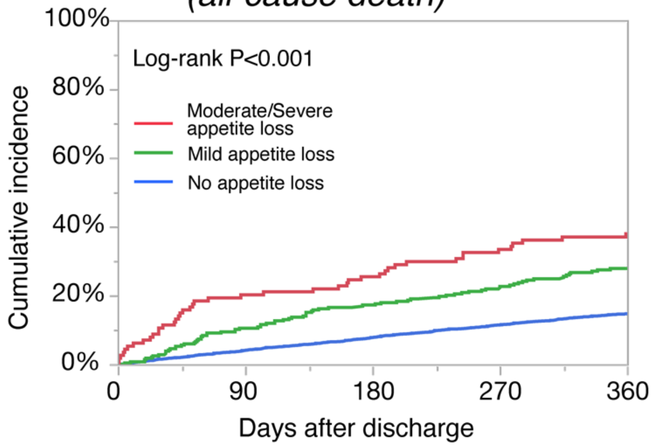


HR (Moderate/Severe vs No appetite loss): 3.20, 95% CI: 2.35-4.36
HR (Mild vs No appetite loss): 2.05, 95% CI: 1.61-2.60

| | 0-day | 30-days | 180-days | 1-year |
|---|---|---|---|---|
| Moderate/Severe appetite loss | | | | |
| N of patients with event | | 12 | 29 | 43 |
| N of patients at risk | 115 | 102 | 85 | 67 |
| Cumulative incidence | | 10.5% | 25.4% | 37.9% |
| Mild appetite loss | | | | |
| N of patients with event | | 8 | 49 | 78 |
| N of patients at risk | 290 | 280 | 232 | 185 |
| Cumulative incidence | | 2.8% | 17.2% | 27.8% |
| No appetite loss | | | | |
| N of patients with event | | 46 | 242 | 457 |
| N of patients at risk | 3123 | 3064 | 2821 | 2320 |
| Cumulative incidence | | 1.5% | 7.8% | 15.0% |

## *(B) CV death*

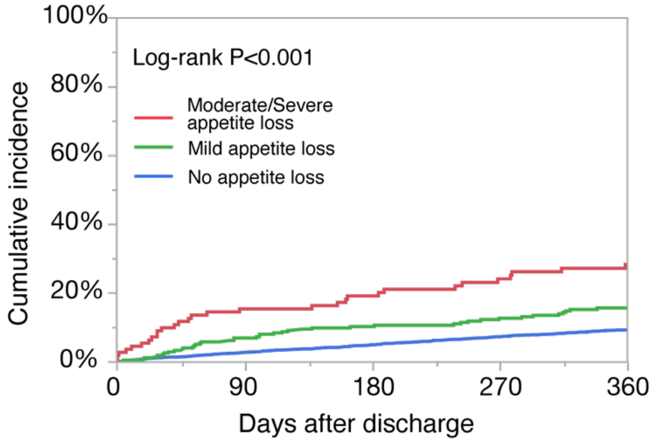


HR (moderate/Severe vs No appetite loss): 3.75, 95% CI: 2.58-5.43
HR (Mild vs No appetite loss): 1.79, 95% CI: 1.29-2.48

| | 0-day | 30-days | 180-days | 1-year |
|---|---|---|---|---|
| Moderate/Severe appetite loss | | | | |
| N of patients with event | | 10 | 21 | 30 |
| N of patients at risk | 115 | 104 | 86 | 68 |
| Cumulative incidence | | 8.9% | 19.0% | 28.1% |
| Mild appetite loss | | | | |
| N of patients with event | | 5 | 28 | 41 |
| N of patients at risk | 290 | 281 | 233 | 185 |
| Cumulative incidence | | 1.7% | 10.1% | 15.5% |
| No appetite loss | | | | |
| N of patients with event | | 33 | 147 | 271 |
| N of patients at risk | 3123 | 3064 | 2821 | 2373 |
| Cumulative incidence | | 1.1% | 4.8% | 9.1% |

## *(C) non-CV death*

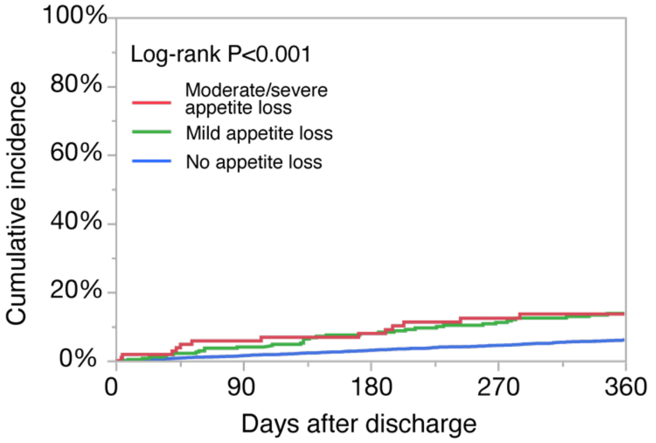


HR (Moderate/Severe vs No appetite loss): 2.44, 95% CI: 1.39-4.29
HR (Mild vs No appetite loss): 2.37, 95% CI: 1.65-3.40

| | 0-day | 30-days | 180-days | 1-year |
|---|---|---|---|---|
| Moderate/Severe appetite loss | | | | |
| N of patients with event | | 3 | 9 | 13 |
| N of patients at risk | 115 | 104 | 86 | 68 |
| Cumulative incidence | | 1.8% | 7.9% | 13.6% |
| Mild appetite loss | | | | |
| N of patients with event | | 6 | 25 | 35 |
| N of patients at risk | 290 | 281 | 233 | 185 |
| Cumulative incidence | | 1.1% | 7.9% | 13.7% |
| No appetite loss | | | | |
| N of patients with event | | 21 | 99 | 177 |
| N of patients at risk | 3123 | 3064 | 2821 | 2373 |
| Cumulative incidence | | 0.4% | 3.0% | 6.0% |

## *(D) HF hospitalization*

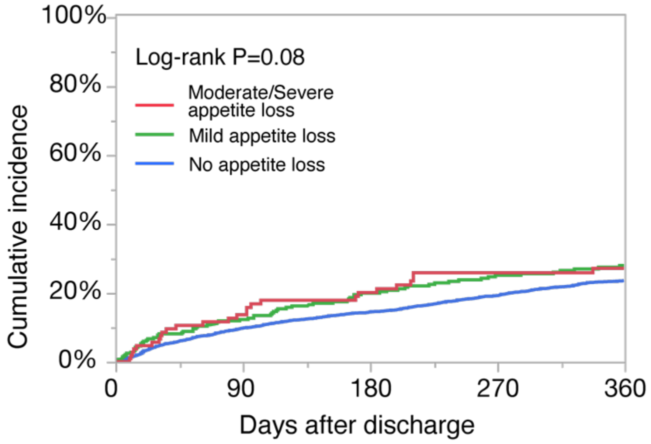


HR (Moderate/Severe vs No appetite loss): 1.24, 95% CI: 0.84-1.84
HR (Mild vs No appetite loss): 1.28, 95% CI: 1.01-1.64

| | 0-day | 30-days | 180-days | 1-year |
|---|---|---|---|---|
| Moderate/Severe appetite loss | | | | |
| N of patients with event | | 6 | 20 | 26 |
| N of patients at risk | 115 | 99 | 75 | 58 |
| Cumulative incidence | | 5.6% | 19.9% | 26.9% |
| Mild appetite loss | | | | |
| N of patients with event | | 22 | 53 | 71 |
| N of patients at risk | 290 | 262 | 195 | 147 |
| Cumulative incidence | | 7.7% | 19.7% | 27.7% |
| No appetite loss | | | | |
| N of patients with event | | 143 | 435 | 689 |
| N of patients at risk | 3123 | 2940 | 2480 | 1857 |
| Cumulative incidence | | 4.6% | 14.4% | 23.5% |
