# Supplementary material for: Appetite loss at discharge from acute decompensated heart failure: Observation from KCHF registry
Source: PLoS One. 2022 May 5;17(5):e0267327. doi: 10.1371/journal.pone.0267327 (PMC9071124; doi:10.1371/journal.pone.0267327)
Supplement: S1 Table — (PDF) [file pone.0267327.s002.pdf]

S2 Table. Patient characteristics without ACE-Is/ARB use.

	Appetite loss (-)			Appetite loss (+)		
	ACE-I/ARB (+)	ACE-I/ARB (-)	P	ACE-I/ARB (+)	ACE-I/ARB (-)	P
Age	78 (69-85)	82 (74-87)	<0.001	82 (73-87)	84 (78-89)	0.02
Age ≧80	843 (45.4)	725 (57.3)	<0.001	111 (57.8)	146 (68.5)	0.03
Male	1098 (59.2)	663 (52.4)	<0.001	97 (50.5)	95 (44.6)	0.23
BMI≦22	750 (41.8)	587 (49.2)	<0.001	98 (54.8)	116 (59.5)	0.35
history of HF	623 (33.9)	451 (36.3)	0.17	69 (38.1)	108 (51.7)	0.007
Anemia	1123 (60.5)	910 (72.1)	<0.001	126 (66.6)	166 (77.9)	0.006
Alb<3.0mg/dL	189 (10.4)	188 (15.4)	<0.001	34 (18.5)	40 (19.6)	0.78
eGFR<30	355 (19.1)	421 (33.3)	<0.001	55 (28.7)	83 (39.2)	0.03
Na<135	177 (9.6)	171 (13.6)	<0.001	21 (10.9)	39 (18.5)	0.03
CRP>1.0	369 (22.2)	339 (29.1)	<0.001	79 (64.8)	105 (75.0)	0.07
EF<40%	769 (41.5)	383 (30.4)	<0.001	79 (41.4)	63 (29.6)	0.01

ACE-I=angiotensin-converting enzyme inhibitor, ARB=angiotensin II receptor blocker, BMI=body mas. Index, CRP=C-reactive protein, eGFR=estimated glomerular filtration rate, EF=ejection fraction, HF=heart failure
