# Supplementary material for: Appetite loss at discharge from acute decompensated heart failure: Observation from KCHF registry
Source: PLoS One. 2022 May 5;17(5):e0267327. doi: 10.1371/journal.pone.0267327 (PMC9071124; doi:10.1371/journal.pone.0267327)
Supplement: S2 Table — (PDF) [file pone.0267327.s003.pdf]

S4 Table. Subgroup analysis for the effect of appetite loss on the primary outcome measure

	Appetite loss (+)	(%)	Appetite loss (-)	(%)	Unadjusted HR	Unadjusted 95%CI	Unadjusted P	Adjusted HR	Adjusted 95%CI	Adjusted P	Interaction P
<80 years	92/257	37.1	321/1568	21.0	2.55	1.72-3.79	<0.001	1.68	1.08-2.61	0.02	0.39
>=80 years	30/148	20.6	136/1555	8.9	2.01	1.60-2.54	<0.001	1.75	0.33-2.30	<0.001	
eGFR<30 mL/min/1.73m^2	52/143	37.7	159/747	21.6	2.51	1.93-3.26	<0.001	1.92	1.32-2.80	<0.001	0.54
eGFR>=30 mL/min/1.73m^2	70/255	28.0	288/2339	12.6	1.98	0.45-2.71	<0.001	1.62	1.18-2.21	0.003	
Ambulatory	64/260	25.5	276/2533	11.1	2.55	1.94-3.34	<0.001	2.15	1.59-2.90	<0.001	0.02
Non-ambulatory	58/140	42.2	176/560	32.5	1.45	1.08-1.95	0.01	1.36	0.94-1.96	0.10	
CRP >=1.0 mg/dL	61/133	46.5	152/708	22.0	2.11	1.59-2.80	<0.001	2.12	1.45-3.08	<0.001	0.19
CRP <1.0 mg/dL	59/249	24.5	262/2116	12.7	2.63	1.95-3.54	<0.001	1.48	1.07-2.05	0.02	
ACE-Is/ARBs (+)	41/192	21.9	200/1857	11.0	2.17	1.55-3.04	<0.001	1.41	0.94-2.09	0.09	0.27
ACE-Is/ARBs (-)	81/213	39.2	257/1266	20.8	2.19	1.71-2.82	<0.001	1.96	1.46-2.62	<0.001	
β blocker (+)	71/251	29.1	264/2112	12.8	2.56	1.97-3.33	<0.001	1.76	1.30-2.39	0.003	0.95
β blocker (-)	51/154	34.0	193/1011	19.7	2.01	1.47-2.73	<0.001	1.86	1.30-2.66	0.007	
Medication >=10	44/148	29.7	196/1166	16.8	2.02	1.46-2.81	<0.001	1.58	1.08-2.31	0.02	0.71
Medication <10	74/230	32.2	235/1823	12.9	2.89	2.22-3.75	<0.001	1.95	1.42-2.98	<0.001	

ACE-I=angiotensin-converting enzyme inhibitor, ARB=angiotensin II receptor blocker, eGFR=estimated glomerular filtration rate
