# Supplementary material for: Appetite loss at discharge from acute decompensated heart failure: Observation from KCHF registry
Source: PLoS One. 2022 May 5;17(5):e0267327. doi: 10.1371/journal.pone.0267327 (PMC9071124; doi:10.1371/journal.pone.0267327)
Supplement: S1 File — (PDF) [file pone.0267327.s004.pdf]

S1 Supplementary Methods

Definitions for the baseline factors

The definitions of the baseline factors in KCHF registry were according to the previous report [1][2]. Atrial fibrillation (AF) included paroxysmal AF, persistent AF, and permanent AF. Hypertension was defined as receiving anti-hypertensive drugs or systolic blood pressure ≥140 mmHg or diastolic blood pressure ≥90 mmHg. Diabetes mellitus was defined as treatment with oral hypoglycemic agents and/or insulin, prior clinical diagnosis of diabetes, glycated hemoglobin level ≥6.5 %, casual blood glucose level ≥200 mg/dl, or fasting blood glucose level ≥126 mg/dl. The presence of COPD was determined clinically by local investigators, based on history, clinical presentation, previous examinations, and medications, recorded as COPD in the case report form at enrolment. Underlying heart disease was defined as the most likely cause of structural or functional cardiac disorders, among those only one category was chosen. The underlying heart disease was classified as (i) coronary artery disease, (ii) hypertensive heart disease, (iii) cardiomyopathy, (iv) valvular heart disease, (v) other heart diseases. Coronary artery disease was defined as acute coronary syndrome (ACS), old myocardial infarction, or prior PCI/CABG. ACS was defined as the range of myocardial ischemic states that includes ST-elevated myocardial infarction, non-ST elevated myocardial infarction, or unstable angina. Primary cardiomyopathy was classified as hypertrophic cardiomyopathy, dilated cardiomyopathy, and dilated phase of hypertrophic cardiomyopathy. Valvular heart disease was classified as moderate to severe aortic stenosis, aortic regurgitation, mitral stenosis, mitral regurgitation (excluding functional mitral regurgitation), tricuspid regurgitation, and prosthetic valve dysfunction. As the valvular heart disease, we chose only one category, which seemed to have most closely related to acute heart failure. Other heart diseases included other cardiomyopathy, arrhythmia (bradycardia or tachycardia), congenital heart disease, and constrictive pericarditis. Other cardiomyopathy included arrhythmogenic right ventricular dysplasia, takotsubo cardiomyopathy, cardiac sarcoidosis, cardiac amyloidosis, left ventricular noncompaction, drug-induced cardiomyopathy, pacemaker induced cardiomyopathy, mitochondrial cardiomyopathy, peripartum cardiomyopathy, alcoholic cardiomyopathy, beriberi heart, and others. Chronic kidney disease was defined as an estimated glomerular filtration rate (eGFR) <60 mL/min per 1.73 m2 at admission. The eGFR was calculated using the equation for the Japanese population: eGFR = 194 × (serum creatinine–1.094) × (age–0.287) × 0.739 (for women). Geriatric nutritional risk index was calculated as follows: 14.89 × (serum albumin[g/dL]) + 41.7 × (body weight/ ideal body weight). Ideal body weight was calculated as follows: 22 ×(height[m]^2). Patient's ADL level was assessed on four level: (1) ambulatory (patients who can walk by themselves), (2) use wheelchair (outdoor only), (3) use wheelchair (outdoor and indoor), (4) bedridden.

The approval number of each participant site

Kyoto University Hospital Kyoto University Graduate School of Medicine (approval number: E2311), Shiga General Hospital (approval number: 20141120-01), Tenri Hospital (approval number: 640), Kobe City Medical Center General Hospital (approval number: 14094), Hyogo Prefectural Amagasaki General Medical Center (approval number: Rinri 26-32), National Hospital Organization Kyoto Medical Center (approval number: 14-080), Mitsubishi Kyoto Hospital (approved 11/12/2014), Okamoto Memorial Hospital (approval number: 201503), Japanese Red Cross Otsu Hospital (approval number: 318), Hikone Municipal Hospital

(approval number: 26-17), Japanese Red Cross Osaka Hospital (approval number: 392), Shimabara Hospital (approval number: E2311), Kishiwada City Hospital (approval number: 12), Kansai Electric Power Hospital (approval number: 26-59), Shizuoka General Hospital (approval number: Rin14-11-47), Kurashiki Central Hospital (approval number: 1719), Kokura Memorial Hospital (approval number: 14111202), Kitano Hospital (approval number: P14-11-012), and Japanese Red Cross Wakayama Medical Center (approval number: 328).

Reference

1. Yamamoto E, Kato T, Ozasa N, Yaku H, Inuzuka Y, Tamaki Y, Kitai T, Morimoto T, Taniguchi R, Iguchi M, Kato M, Takahashi M, Jinnai T, Ikeda T, Nagao K, Kawai T, Komasa A, Nishikawa R, Kawase Y, Morinaga T, Kawashima T, Motohashi Y, Kawato M, Toyofuku M, Sato Y, Kuwahara K, Shioi T, Kimura T. Kyoto Congestive Heart Failure (KCHF) study: rationale and design. *ESC Hear Fail*. 2017;4:216–223.
2. Yaku H, Ozasa N, Morimoto T, Inuzuka Y, Tamaki Y, Yamamoto E, Yoshikawa Y, Kitai T, Taniguchi R, Iguchi M, Kato M, Takahashi M, Jinnai T, Ikeda T, Nagao K, Kawai T, Komasa A, Nishikawa R, Kawase Y, Morinaga T, Su K, Kawato M, Sasaki K, Toyofuku M, Furukawa Y, Nakagawa Y, Ando K, Kadota K, Shizuta S, Ono K, Sato Y, Kuwahara K, Kato T, Kimura T, KCHF Study Investigators. Demographics, Management, and In-Hospital Outcome of Hospitalized Acute Heart Failure Syndrome Patients in Contemporary Real Clinical Practice in Japan - Observations From the Prospective, Multicenter Kyoto Congestive Heart Failure (KCHF) Registry. *Circ J*. 2018;82:2811–2819.

Clinical event committee

Mitsuaki Kawato, Nishi Kobe Medical Center

Mamoru Toyofuku, Japanese Red Cross Wakayama Medical Center
