# Supplementary material for: Appetite loss at discharge from acute decompensated heart failure: Observation from KCHF registry
Source: PLoS One. 2022 May 5;17(5):e0267327. doi: 10.1371/journal.pone.0267327 (PMC9071124; doi:10.1371/journal.pone.0267327)
Supplement: Attachment — Submitted filename: rebuttal_appetite_1223.docx [file pone.0267327.s005.docx]

 Review Comments to the Author

Reviewer #1: A paper of Yamamoto et al. presents results of an observational study which included over 3 thousand patients with acute heart failure. The study population and follow-up time (mean 1-2 years) are the great advantages of the study. Methods and results are clearly described. Unfortunately, discussion is very simplistic with only a superficial analysis of potential mechanisms linking appetite loss and heart failure.

*Thank you so much for the important comment. Actually, the analysis is very simple in this study. KCHF study is not just focused on the nutrition and heart failure, therefore we didn’t collect many important factors, such as the change of body weight, or any objective evaluation measure about appetite loss (CNAQ-J, MNA etc.) and cannot draw any conclusion about the causal relationship between appetite, nutrition status, and prognosis. We would like to avoid overstating the result and just show that the patient with appetite loss at hospital discharge was associated with poor outcome. Based on this situation, we added several sentences in discussion part.*

The current guidelines mention frailty, cachexia, and sarcopenia, as well as iron deficiency.^12^ Efficient caloric intake and nutrition supplementation is necessary to solve these problems, and it is also important to address loss of appetite. Recently, Anamorelin has been used for cancer cachexia. Anamorelin exerts its effects by acting on the ghrelin receptor, GHS-R1a. GHS-R1a is distributed in many tissues and is involved in the release of growth hormone in the pituitary gland and the enhancement of appetite. Anamorelin may have muscle mass and weight gain effects by promoting GH secretion and increasing appetite through activation of GHS-R1a. Although Anamorelin is contraindicated in heart failure patients because of its negative effect for cardiac function, pharmaceutical intervention as well as nutritional intervention may be an option in the future.

Moreover, Authors haven’t referred any original papers concerning the issue of appetite loss and anorexia/cachexia syndrome in heart failure (and in the past years there were at least few important papers published).

*Thank you so much for your constructive comment. We added several references.*

First paragraph of the discussion is in the fact repeated results’ summary which, in my opinion, is needless.

*Thank you so much for the comment. We deleted summary in discussion.*

1. Phrase “ambulatory status” in the abstract is unclear – did the Authors meant “ambulatory” vs “hospitalized” or NYHA class? Then in the result section again is an information that “Ambulatory patients were less frequent in the appetite loss group” – it should a status at hospital discharge so what is the “non-ambulatory status”? I think it should be defined.

*Thank you so much for the comment.* *We defined ambulatory status as patients who can walk by themselves. In this study, we assessed patient’s ADL on four level: (1) ambulatory (patients who can walk by themselves), (2) use wheelchair (outdoor only), (3) use wheelchair (outdoor and indoor), (4) bedridden. “Ambulatory status” means patients with highest ADL level among them. We added definition in supplementary appendix.*

2. Where are the results of subgroup analysis depending on “activity of daily living”?

*Thank you so much for the comment. As mentioned above, “ambulatory” means ADL status. “ambulatory” vs “non-ambulatory” (the third in supplementary table 1) correspond the result of subgroup analysis depending on ADL.*

3. “Neither loss nor increase of appetite is common as a side effect of ACE-Is/ARBs, but the positive link between the absence of ACE-Is/ARB use and appetite loss is presumed to be the reflection of renal function and poor general conditions of the patients with appetite loss.” – there is not direct data presented in the study which could support the thesis.

*Thank you so much for the constructive comment. We evaluated whether general condition, including renal function is worse in patients without ACE-Is/ARB use. We have added the Characteristics of patients without ACE-Is/ARB use in the Supplementary Table 1.*

|  |  | Appetite loss (-) |  |  |  | Appetite loss (+) |  |
| --- | --- | --- | --- | --- | --- | --- | --- |
|  | ACE-I/ARB (+) | ACE-I/ARB (-) | P |  | ACE-I/ARB (+) | ACE-I/ARB (-) | P |
| Age | 78 (69-85) | 82 (74-87) | <0.001 |  | 82 (73-87) | 84 (78-89) | 0.02 |
| Age ≧80 | 843 (45.4) | 725 (57.3) | <0.001 |  | 111 (57.8) | 146 (68.5) | 0.03 |
| Male | 1098 (59.2) | 663 (52.4) | <0.001 |  | 97 (50.5) | 95 (44.6) | 0.23 |
| BMI≦22 | 750 (41.8) | 587 (49.2) | <0.001 |  | 98 (54.8) | 116 (59.5) | 0.35 |
| hx of HF | 623 (33.9) | 451 (36.3) | 0.17 |  | 69 (38.1) | 108 (51.7) | 0.007 |
| Anemia | 1123 (60.5) | 910 (72.1) | <0.001 |  | 126 (66.6) | 166 (77.9) | 0.006 |
| Alb<3.0mg/dL | 189 (10.4) | 188 (15.4) | <0.001 |  | 34 (18.5) | 40 (19.6) | 0.78 |
| eGFR<30 | 355 (19.1) | 421 (33.3) | <0.001 |  | 55 (28.7) | 83 (39.2) | 0.03 |
| Na<135 | 177 (9.6) | 171 (13.6) | <0.001 |  | 21 (10.9) | 39 (18.5) | 0.03 |
| CRP>1.0 | 369 (22.2) | 339 (29.1) | <0.001 |  | 79 (64.8) | 105 (75.0) | 0.07 |
| HFrEF | 769 (41.5) | 383 (30.4) | <0.001 |  | 79 (41.4) | 63 (29.6) | 0.01 |

*Patients without ACE-I/ARB were older, had lower BMI and worse renal function compared to patients with ACE-I/ARB in both patients with and without appetite loss.*

4. Decreased serum albumin and hemoglobin concentration are not just a sign of nutrition status but reflect advanced pathological changes in heart failure, including i.a. liver and renal failure, iron deficiency

*Thank you so much for the comment. We agree with author’s comment. We added the sentence below in discussion part.*

“Decreased serum albumin is not just a sign of nutrition status but reflect congestion and advanced pathological changes in liver due to chronic liver congestion. Anemia in patients with heart failure, as well as hypoalbuminemia, is not only a reflection of nutritional status, but also a result of chronic inflammation caused by heart failure itself, concomitant renal dysfunction and reduced erythropoietin production and sensitivity, as well as suppression of hematopoietic stem cell function by ACE inhibitors and angiotensin II receptor blockers.”

Reviewer #2: This is a retrospective analysis from the Kyoto Congestive Heart Failure registry focusing on the prevalence and prognostic impact of appetite loss in 3,528 patients with acute heart failure. As one might expect, appetite loss at discharge was associated with worse clinical status and a worse 1-year outcome. The study is original in that it explores appetite loss in this setting. On the other hand, it has the limitations of a retrospective analysis on a non-dedicated database, namely the lack of relevant information such as food intake etc. Study limitations are correctly acknowledged by the Authors. Please find below some other comments.

Patients were not selected based on their cognitive status, and assessment of appetite loss (as well as its classification as mild, moderate or severe) relied exclusively on patient report. Food intake and calories per day could have been easily checked in hospitalized patients, and would have provided objective metrics for analysis.

*Thank you for the constructive comment. We would like to collect these quantitative data in next study.*

Please specify more clearly what you mean by "ambulatory status" (patients treated with iv diuretics without hospitalization?)

*Thank you for the comment. We defined ambulatory status as patients who can walk by themselves. In this study, we assessed patient’s ADL on four level: (1) ambulatory (patients who can walk by themselves), (2) use wheelchair (outdoor only), (3) use wheelchair (outdoor and indoor), (4) bedridden. “Ambulatory status” means patients with highest ADL level among them. We added definition in supplementary appendix.*

Apart from medications at discharge, you should have considered also the start of drugs from admission to discharge, as starting multiple therapies could reduce patient appetite.

*Thank you for pointing out important point. We added the sentence below in limitation part.*

*“Forth, newly initiated medication during hospitalization may affect appetite, however appetite date was only available at discharge, and the association with those medication is unknown.”*

Table 2 reports only univariable predictors. Please provide the full list of variables evaluated as possible univariable predictors. It is important to understand if relevant variables such as the length of hospitalization were taken into account. The list of univariable predictors is rather short, with several arbitrarily dichotomized variables.

*Thank you for the comment.* *We assessed clinical and laboratory data shown in Table 1, and choose 19 factors with P value < 0.1 by univariate analysis. We added the underlined part in method section.*

A multivariable logistic regression model was developed to identify clinical characteristics, and laboratory data and medications at discharge that were independently associated with the presence of appetite loss using 19 factors with P value < 0.1 by univariate analysis **extracted from the variables listed in Table 1.”**

In the abstract, please specify the variables included in the multivariable model.

*Thank you for the comment. We added the underlined part in abstract.*

“In the multivariable logistic regression analysis **using 19 clinical and laboratory factors with P value < 0.1 by univariate analysis**, BMI…”

The approval numbers by the many centers (page 12) could be moved　to the supplementary material.

*Thank you for the comment.* *We moved the individual approval numbers to supplementary appendix.*

The manuscript would benefit from linguistic revision.

*Thank you for the constructive comment. The revised manuscript has been edited by native speaker.*
