# Supplementary material for: Appetite loss at discharge from acute decompensated heart failure: Observation from KCHF registry
Source: PLoS One. 2022 May 5;17(5):e0267327. doi: 10.1371/journal.pone.0267327 (PMC9071124; doi:10.1371/journal.pone.0267327)
Supplement: Attachment — Submitted filename: comment for reviewer_R2_final.docx [file pone.0267327.s006.docx]

Reviewer #1: The revised manuscript replies to most but not all comments of the previous review. Authors significantly improved the discussion section and clarified some ambiguities. Nevertheless, in my opinion, some parts still needs explanation or completion.

We appreciate the time and effort that you have dedicated to providing your valuable feedback on my manuscript. We revised the manuscript again according to your comments.

The definition of “ambulatory status” should be placed in the main text body not in the supplement.

Thank you so much for the comment. We moved the definition of “ambulatory status” in the supplement to main text.

A statement “However, there was no study focused on “appetite loss,” which is a subjective symptom in patients with heart failure. “ is untrue – there are studies concerned on appetite loss in patient with heart failure and Authors have been already asked to quote these in the discussion.

Thank you so much for the comment. Based on your comment, we have revised the sentence as follows.

***P3 L15 Although in the past years there were at least few important papers published concerning the issue of appetite loss and anorexia/cachexia syndrome in heart failure [PMID:*** ***9107242, PMID: 25704364, PMID:*** ***34757487, PMID:*** ***24347122, PMID:*** ***26865478], there is still scarcity in the studies focused on “appetite loss,” a subjective symptom in patients with heart failure [PMID:*** ***24347122, PMID:*** ***26865478].***

There are sentences which need rephrasing:
- “We assessed 3528 patients who were alive at discharge for whom appetite and had follow-up data were available.”

Thank you so much for the comment. Based on your comment, we have revised the sentence as follows.

***P2 L7 We assessed 3528 patients alive at discharge, and for whom appetite and follow-up data were available.***

- “In the multivariable logistic regression analysis using 19 clinical and laboratory factors with P value < 0.1 by univariate analysis, BMI < 22 kg/m2 (odds ratio (OR): 1.57, 95% confidence interval (CI): 1.11-2.24, P=0.01), CRP >1.0mg/dL (OR: 1.49, 95%CI: 1.04-2.14, P=0.03), and presence of edema at discharge (OR: 4.30, 95%CI: 2.99-6.22, P<0.001) were positively associated with appetite loss at discharge, whereas ambulatory status (OR: 0.57, 95%CI: 0.39-0.83, P=0.004) and the use of ACE-I/ARB (OR: 0.70, 95% CI: 0.50-0.99, P=0.04) were negatively associated with appetite loss.” Phrase “positive/negative association” when odds ratio are used is a little bit unfortunate because it is not a correlation but risk increase/decrease.

Thank you so much for the comment. Based on your comment, we have revised the sentence as follows.

***P2 L9 In the multivariable logistic regression analysis using 19 clinical and laboratory factors with P value < 0.1 by univariate analysis, BMI < 22 kg/m^2^ (odds ratio (OR): 1.57, 95% confidence interval (CI): 1.11-2.24, P=0.01), CRP >1.0mg/dL (OR: 1.49, 95%CI: 1.04-2.14, P=0.03), and presence of edema at discharge (OR: 4.30, 95%CI: 2.99-6.22, P<0.001) were associated with an increased risk of appetite loss at discharge, whereas ambulatory status (OR: 0.57, 95%CI: 0.39-0.83, P=0.004) and the use of ACE-I/ARB (OR: 0.70, 95% CI: 0.50-0.99, P=0.04) were related to decrease risk in the presence of appetite loss.***

- “The details have been previously descrived.” – please correct a typing mistake

Thank you so much for the comment. Based on your comment, we have revised the sentence as follows.

***P5 L9 The details have been previously described.***

- “When we consider the cause of appetite loss, this result is clinically important, because there is no argument about the association between the amount of activity, nutritional status, and the presence of appetite loss.” – the sentence is either unclear or false. There are many links between amount of activity, nutritional status and appetite loss.

Thank you so much for the comment. As you pointed out, we also think there are many links between these factors and appetite loss and the result of this study are reasonable. We revised the sentence more simply.

***P19 L14 When we consider the cause of appetite loss, there is no argument about the association between the amount of activity, nutritional status, and the presence of appetite loss.***

- “In addition, low output due to heart failure leads intestinal edema and dysfunction.
Congestion and hypoalbuminemia due to heart failure also leads intestinal edema and
dysfunction, resulting in loss of appetite.” or “leads to” ?

Thank you so much for the comment. Based on your comment, we have revised the sentence as follows.

***P20L5 In addition, low output due to heart failure leads to intestinal edema and dysfunction. Congestion and hypoalbuminemia due to heart failure also leads to intestinal edema and dysfunction, resulting in loss of appetite.***
