# Supplementary material for: Appetite loss at discharge from acute decompensated heart failure: Observation from KCHF registry
Source: PLoS One. 2022 May 5;17(5):e0267327. doi: 10.1371/journal.pone.0267327 (PMC9071124; doi:10.1371/journal.pone.0267327)
Supplement: Attachment — Submitted filename: Response appetite_220319.docx [file pone.0267327.s007.docx]

**Response:**

**We thank the reviewer for detailed assessment and positive comments. We have revised the manuscript according to your suggestions.**

Reviewer #1: The majority of concerns raised in the previous review were addressed. However, there are a few questions left.

I still have doubts about the following conclusion:
“When we consider the cause of appetite loss, because there is no argument about the association between the amount of activity, nutritional status, and the presence of appetite loss.”
It stands in the opposition to the results: BMI < 22 kg/m2 increased the risk of appetite loss incidence (OR: 1.57, 95% CI: 1.11-2.24, P=0.01), whereas ambulatory status (OR: 0.57, 95% CI: 0.39-0.83, P=0.004) decreased. BMI reflect nutritional status, ambulatory status is related to higher activity.

**Response:**

**Thank you for the important comments. As you pointed out, poor nutritional status increased the risk of appetite loss incidence, whereas higher activity decreased the risk of appetite loss incidence. We have revised the manuscript as follows:**

**“BMI < 22 kg/m^2^, which reflects poor nutritional status, increased the risk of appetite loss incidence, whereas ambulatory status, which is related to higher activity, decreased the risk in the presence of appetite loss.” (Page 20, Lines 4-6)**

Authors still used the phrase “positively/negatively associated” in the context of risk assessment:
“In the multivariable logistic regression analysis, BMI < 22 kg/m2 (OR: 1.57, 95% CI: 1.11-
2.24, P=0.01), CRP > 1.0mg/dL (OR: 1.49, 95% CI: 1.04-2.14, P=0.03), and the presence of edema at discharge (OR: 4.30, 95% CI: 12.99-6.22, P<0.001) were positively associated with appetite loss at discharge, whereas ambulatory status (OR: 0.57, 95% CI: 0.39-0.83, P=0.004) and the use of ACE-I/ARB (OR:0.70, 95% CI: 0.50-0.99, P=0.04) were negatively associated with appetite loss (Table 2).” (page 14)

**Response:**

**We appreciate your comments. We have deleted the phrases “positively/negatively associated”. We have changed these as follows:**

**“In the multivariable logistic regression analysis, BMI < 22 kg/m^2^ (OR: 1.57, 95% CI: 1.11-2.24, P=0.01), CRP >1.0mg/dL (OR: 1.49, 95%CI: 1.04-2.14, P=0.03), and presence of edema at discharge (OR: 4.30, 95%CI: 2.99-6.22, P<0.001) were associated with an increased risk of appetite loss at discharge, whereas ambulatory status (OR: 0.57, 95%CI: 0.39-0.83, P=0.004) and the use of ACE-I/ARB (OR: 0.70, 95% CI: 0.50-0.98, P=0.04) were related to a decreased risk in the presence of appetite loss (Table 2)”. (Page 14 Lines 2-7)**

There is a typing mistake in the abstract:
“were related to decrease risk in the presence of appetite loss” instead of: “were related to decreased risk in the presence of appetite loss

**Response:**

**We have corrected the typing mistake. We appreciate your carful assessment.**
